# Promoting adult health: the neurophysiological benefits of watering plants and engaging in mental tasks within designed environments

**DOI:** 10.1186/s40359-023-01362-5

**Published:** 2023-10-06

**Authors:** Ahmad Hassan, Zhang Deshun

**Affiliations:** College of Architecture and Urban Planning, Tongji University, 1239 Siping Road, Shanghai, People’s Republic of China

**Keywords:** Cognitive tasks, Gardening, Neurophysiological advantages, Plant watering, Stress alleviation

## Abstract

**Background:**

Indoor, sedentary lifestyles have disconnected individuals from nature, necessitating interventions to reestablish this bond. Performing horticultural activities, such as watering houseplants, offers a potential solution. This study sought to determine how participating in horticulture activities affected adults’ cognitive and emotional moods.

**Methods:**

We compared the benefits of watering houseplants (a gardening task) to those of standing while performing a computer task (a mental task). Chinese participants, aged 20 to 21 years, were recruited; their physiological and psychological reactions were measured using electroencephalograms, blood pressure assessments, and psychological assessments.

**Results:**

Fifty participants were included. Watering indoor plants significantly reduced blood pressure, without affecting pulse rate. During the plant watering task as opposed to the mental activity, more dramatic different patterns of very high alpha and beta brainwave activity were identified. Participants reported increased happiness following gardening activities.

**Conclusions:**

The findings of this study highlight the substantial relaxation benefits, both mental and physical, associated with the simple act of watering indoor plants.

## Background

In modern society, people spend a significant amount of time indoors; statistics indicate that 95% of an individuals’ activities take place in indoor environments [1]. This sedentary way of life, with insufficient exercise, is prevalent worldwide and is a result of urbanization and advances in technology [2]. Moreover, the ongoing global pandemic of coronavirus disease 2019 has resulted in a rise in the duration of indoor activities, social isolation, and the implementation of solitary precautions within households. These measures have further exacerbated the global trend toward sedentary behavior [3].

Sedentary behavior is defined as engaging in activities when awake and in a sitting, reclining, or lying down position. The energy expended while engaged in sedentary behavior is typically less than 1.5 metabolic equivalents of task [4]. In the United States and Canada, adults spend approximately 9 to 11 h (55–70% of awake hours) being sedentary [5]. In Korea, the average is approximately 8 h [6]. These statistics highlight the challenge individuals face in incorporating sufficient physical activity into their daily routines. Alarmingly, nearly 28% of people in the world who are 18 years or older do not engage in sufficient physical activity [7].

The absence of physical activity and a predominantly sedentary lifestyle have been linked to negative health outcomes, including a decline in physical well-being. Moreover, the progression of adverse impacts on emotional and cognitive well-being, alongside the emergence of conditions that tend to linger for a long time include metabolic syndrome, cardiovascular disease, and type 2 diabetes [8]. Specifically, the various stressors encountered in daily life due to sedentary behavior can exacerbate conditions such as depression, unhealthy habits such as excessive drinking and smoking, social isolation, and disrupted sleep patterns, ultimately resulting in attention deficits and cognitive impairments [9].

Face with an escalating number of people leading sedentary lives, the role of “exposure to nature” or nature therapy emerges as the paramount solution [10]. This approach addresses both the adverse effects of prolonged inactivity and issues such as stress and diminished well-being [11]. Concurrently, scholars are raising concerns about the modern “disconnect from nature,” a consequence of spending more time indoors, isolated from the natural world [12]. Horticultural activities (HAs) are a potent remedy to bridge this gap and restore the bond between humans and nature. Engaging in activities such as gardening, tending to indoor plants, and cultivating green spaces not only promotes physical movement, but also rejuvenates mental faculties [13]. By nurturing plant life, individuals partake in a tangible connection with the environment, fostering a sense of responsibility and appreciation for the natural world. For instance, watering plants, a commonplace HA, symbolizes the nurturing process, offering a tangible way to engage with nature daily. As noted in the literature, such activities can serve as accessible and effective means to reconnect with nature: they offer potential benefits for overall well-being [14].

A regular relationship between people and the natural world has a profound impact on overall well-being and establishes nature as a crucial resource for promoting health [15]. Psycho-Evolutionary Theory, the Theory of Attention Restoration, and the Biophilia Hypothesis are three prominent theoretical frameworks that underscore the intrinsic bond between individuals and the natural environment, as well as the salutary impacts of nature on human well-being [16].A deep connection to nature is linked to a multitude of advantages, which include enhancements in physical health such as decreases in blood pressure, body weight, and cholesterol levels, as well as improved physical function and balance. Additionally, mental well-being is also positively impacted. The potential benefits of engaging in certain activities include stress reduction, favorable changes in mood, better control over impulsive behavior, enhanced self-esteem, as well as improvements in the workings of the mind, including those of attention and memory [17].

Daily exposure to greenery is thought to be crucial for adults to reinforce and develop positive feelings toward nature [18]. A person’s fondness for and curiosity about the natural world are enhanced by early exposure to it [19]. In a study conducted by Bixler et al. (2002) in the United States, it was found that individuals who hold a high regard for wilderness regions tend to experience more enjoyable cognitive experiences when engaging with natural environments [20]. Adults in the United Kingdom who grew up in rural areas tended to have a more positive outlook on the natural world than their urban counterparts, according to research by Hinds and Sparks [21]. These results suggest that early exposure to the natural world shapes a person’s perspective and appreciation of the environment throughout their life. Several other academics and environmentalists have come to the same conclusion: exposure to nature, either directly or indirectly, is crucial for the development of a positive worldview concerning the natural world [22]. Through previous studies [23] and review papers [24], greenery has been shown to have positive health impacts immune function, endocrine function, and the autonomic nervous system. According to Ikei et al. [25] and Park et al. [26], the sight of green plants has been shown to induce a state of physiological and psychological peace simply by stimulating the parasympathetic nervous system. These researchers also noticed that it had a favorable effect on their emotional state. Similarly, after viewing roses, office workers experienced psychological and physiological relaxation in a variety of emotional states [25, 26].

HAs offer dynamic interactions with natural elements, providing an opportunity for active engagement with the environment [27]. These activities involve physical exertion ranging from low to high energy expenditure, engaging various muscles throughout the body [28]; they also contribute to physical, emotional, and cognitive well-being when employed as forms of physical exercise [29]. As a form of “green exercise,” HAs serve as a means to connect individuals with nature, thereby aiding in improving health and prosperity while acting as a preventive measure against sedentary lifestyles and lack of exercise.

A study by Park and colleagues [29] examined how using HAs as a form of physical activity affected cognitive health. The researchers noticed elevated serotonin and tryptophan production metabolisms in HA program participants, indicating elevated sensations of happiness. As a result, brain-derived neurotrophic factor levels significantly increased, suggesting that benefits in memory performance were experienced. Additionally, the visual stimulus supplied by green plants helps to enhance emotional stability and relaxation. This is demonstrated by the fact that blood flow in study participants’ prefrontal lobes slowed and their autonomic nervous system reactions were stabilized [26]. Furthermore, questionnaire-based approaches in various clinical studies have demonstrated the potential of HA programs to enhance attention and emotional well-being [30]. There is a significant knowledge gap because these research have not yet created a solid scientific basis for understanding methods via which HAs produce their positive effects.

A noninvasive wearable device that records an electroencephalogram (EEG) can be used to track macroscopic electrical activity at the brain’s surface [31]. This technique has widespread applications in psychophysiological, cognitive science, cognitive psychology, and clinical medicine, enabling physiological and neurological data to be gathered and evaluated [32]. EEG bands, such as theta, gamma, alpha, and beta waves are employed to analyze different psychophysiological states in participants. Furthermore, recent advances in analysis approaches have enabled secondary parameters to be estimated; power spectrum analysis is one such approach. Estimating secondary parameters facilitates a more comprehensive examination of participants’ attention states. Kim et al. conducted research that showed that, compared to other recreational activities, HAs have a good impact on brain activity, attention, emotional recovery, and other related characteristics, as measured by EEG [33]. Performing HAs has different types of effects on people’s mental health and brain function; however, thus far, the specific outcomes of these effects have not been comprehensively characterized. Also, no studies have investigated the effect that watering plants has on individuals who spend extended periods indoors and who are prone to events—such as academic stress—that can lead to mental issues.

Questions have arisen regarding whether watering plants only offers physical benefits and whether it affects people’s attitudes toward the environment rather than imposing a psychological influence on the relationship between people and the environment. By examining existing research and theories related to environmental psychology, horticultural therapy, and biophilia, this study explores the underlying psychological mechanisms and benefits associated with the act of watering plants in a landscape environment. Understanding the psychology of watering plants in the landscape context has important implications for landscape design, urban planning, and promoting human well-being in modern society. By incorporating water features, interactive plant care, and mindful engagement with plants, landscapes can be designed to support psychological well-being and foster a stronger connection between individuals and their surrounding environment. As a result, the primary The goal of this research was to find out, and build a clearer understanding of, the psychological and physiological relaxation effects among Chinese adults that result from watering indoor plants in comparison to engaging in a mental task.

## Methods

### Study setting

This indoor research study was conducted at the laboratory of the College of Architecture and Urban Planning, Tongji University. The trials were carried out in a single, huge, soundproof room which had white walls, green curtains, and two enormous windows on the north side of the building. The experiment took place in winter and the room was kept warm using an air conditioner. In order to provide the participants with a pleasant working atmosphere free from outside distractions, the room was also kept silent. The experiment was conducted on a large desk, and the measuring tools were kept out of the participants’ reach throughout the study. The experiment was conducted from 8 a.m. to 9 p.m. at a controlled temperature of 25 °C and a light intensity of 500 lx.

### Participants

We recruited 50 Chinese students (mean age, 21.2 ± 0.64 years) who were pursuing majors in landscape architecture, forestry, and horticulture at Tongji University. Advertisements were placed on campus bulletin boards to recruit participants and the advertisements targeted college students who met the following three requirements: aged 19 to 22 years; healthy, without any known physical or mental health concerns; and did not use drugs. Participants were selected at random, and none reported having any type of physical or mental problem in the preceding 3 months. A research stipend of 50 RMB was awarded to each participant after they completed the study. Participants were given a thorough explanation of the experiment before it was performed, and their written informed consent, including permission to have their images posted online, was acquired. Before this experiment was conducted, it was approved by the local Ethics Committee of the College of Architecture and Urban Planning at Tongji University in China.

### Protocol

A general HA (watering indoor plants) was chosen as the plant watering task. A written daily assignment task on a computer—one of the most frequently performed academic tasks—was selected as the mental (control) task as it was comparable to the HA task and required continuous movement. Prior to performing the experiment, a complete presentation of the experimental methodology was given to the participants.

**Fig. 1:**
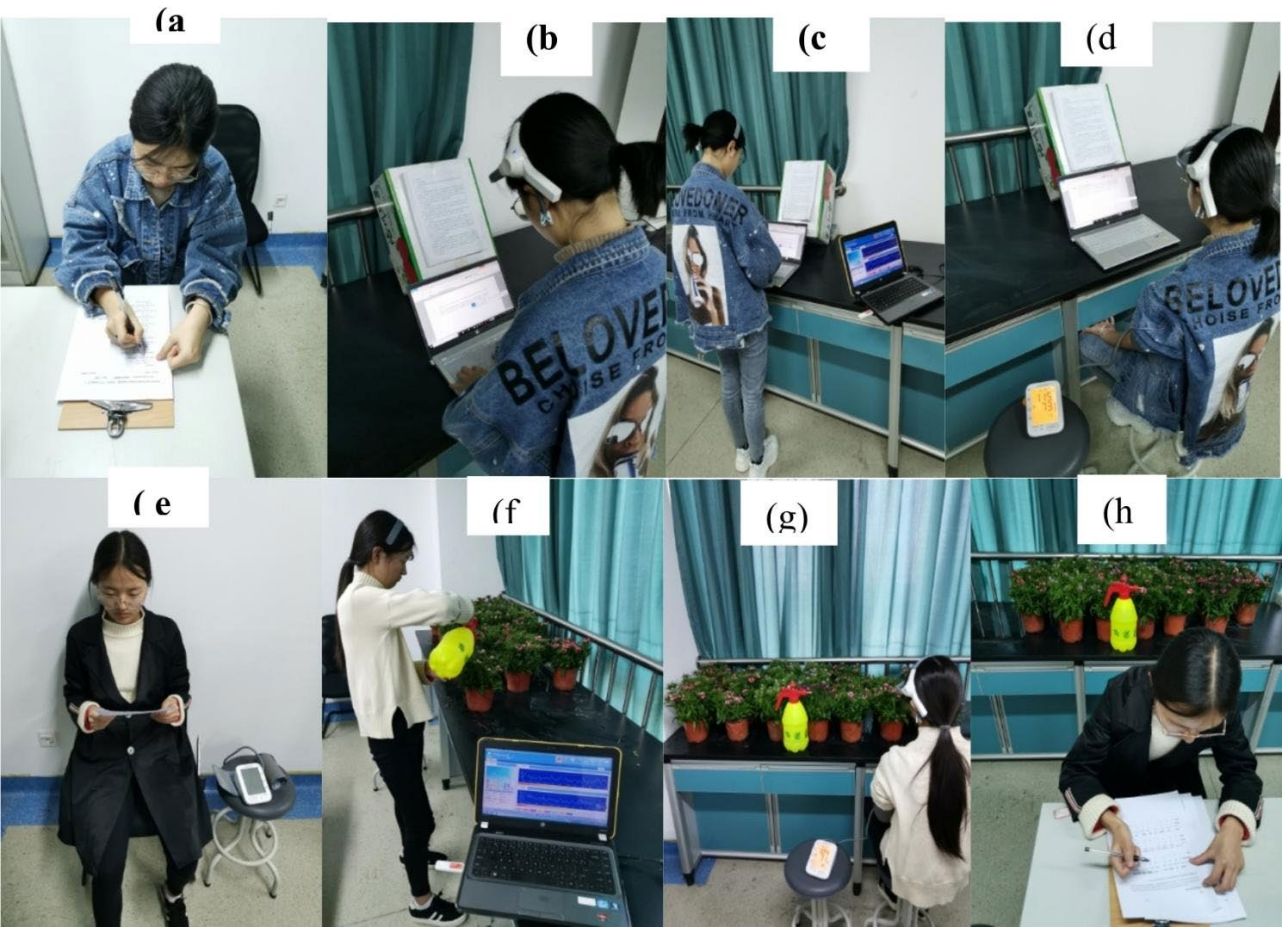
Experimental photographs during both tasks. (**a**) A subject filling pretest questionnaires; (**b**-**c**) a subject performing a mental task; (**d**) blood pressure measurement after the mental task; (**e**) a subject reading the instructions before performing the plant watering task; (**f**-**g**) a subject performing the plant watering task; (**h**) a subject filling post-test questionnaires

Participants were split into two groups of 25 students each, namely the horticulture group and the control group. The horticultural group completed the plant watering task, while the control group completed the mental task. Participants carried out their assigned tasks for 15 min at a large laboratory table against a window with green curtains. Further details on the experimental procedure are shown in Fig. 1.

### Plant watering task

Twenty Dianthus (family, Caryophyllaceae) indoor plants were selected from a local Chinese garden nursery. These plants were chosen based on their aromatic nature and green leaves with multi-colored varieties. The selected plants were of similar size, with a diameter and height ranging from approximately 3 to 5 mm. Participants were required to water the plants manually. Each plant cost 50 RMB.

### Measurements

Participants were instructed and guided on how to complete the pretest questionnaires, which contained questions regarding their age, weight, height, SDM, and the State-Trait Anxiety Inventory (STAI). After completing the pretest questionnaires, each participant was given consent forms with details regarding the experiment. Thereafter, they five minutes of sitting-down rest. Their baseline blood pressure was taken after they had rested, and the EEG headset was affixed to the top of the participant’s head. Each participant subsequently completed their assigned mental or plant watering task for 15 min. The data on subjects’ EEG and blood pressure were gathered throughout the task to determine their physiological reactions. Blood pressure and pulse rate were measured using a sphygmomanometer (Omron HEM-7011; Omron Corporation, China), and electrical brain activity was measured using a NeuroSky MindWave EEG headset (MW001; Beijing Oriental Creation Technology Co., Ltd., China). This headset captures electrical brainwave signals from the frontal lobe, which is above the eye, at the Fp1 position [34]. It is made up of four main parts: a headband, an EEG electrode on a sensor arm, a Bluetooth device, and an ear clip. Two dry sensors are generally used to filter the recorded EEG signals, and a sensor tip is utilized to record electrical impulses in the brain from the Fp1 location. The sensor also records background noise from electrical devices like computers, lightbulbs, outlets, and muscles. The ear clip serves as a ground and a reference point, allowing the ThinkGear chip to filter out electrical noise [35]. In addition to the raw data, the new EEG headset can record high alpha and beta power spectra. A rate of 512 Hz (or once per second) is used to capture raw EEG data. The information is then processed by tiny microchips before being sent as electrical signals to a computer via the Bluetooth device. In this study, we analyzed the raw EEG data of high alpha and beta power units acquired at 1-minute intervals for each task to compare the total 15-minute averages of the two groups. After concluding the task, participants completed the self-reported SDM [36] and STAI [37] to assess the their psychological responses to performing the tasks.

### Statistical analysis

For statistical analysis, we utilized SPSS version 16.0 software (SPSS Inc., Chicago, IL, USA). We used the Wilcoxon signed-rank test to assess psychological data and the paired-samples t-test and repeated-measures analysis of variance to analyze physiological data. A p-value of 0.05 was used in the analysis of both the physiological and psychological data to denote statistical significance.

## Results

**Fig. 2:**
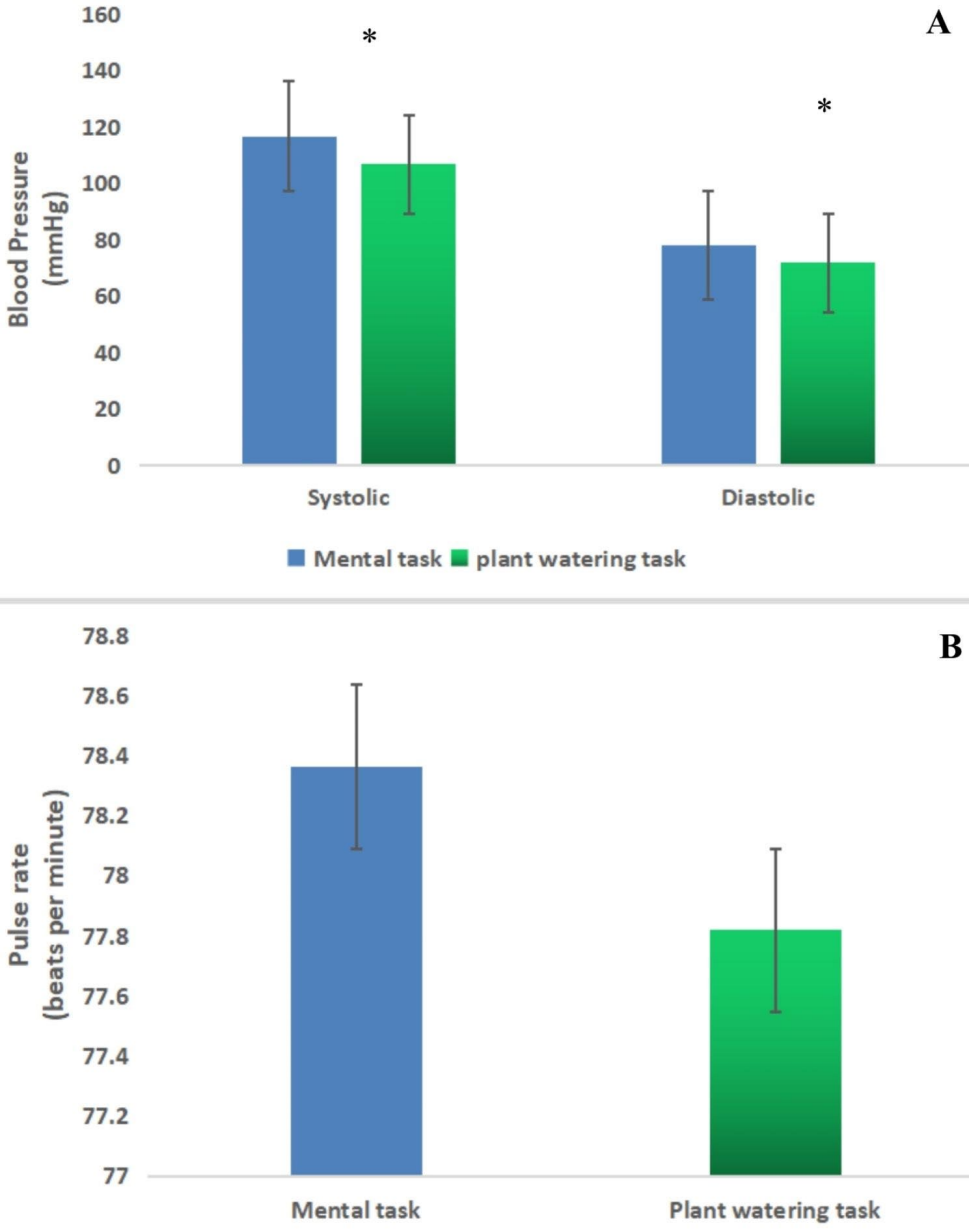
(**A**) Comparison of systolic and diastolic blood pressure measurements after the plant watering task and mental task (control); (**B**) Effect of the plant watering task and mental task (control) on pulse rate. N = 50; mean ± SE; *P < 0.05, paired t-test

**Fig. 3:**
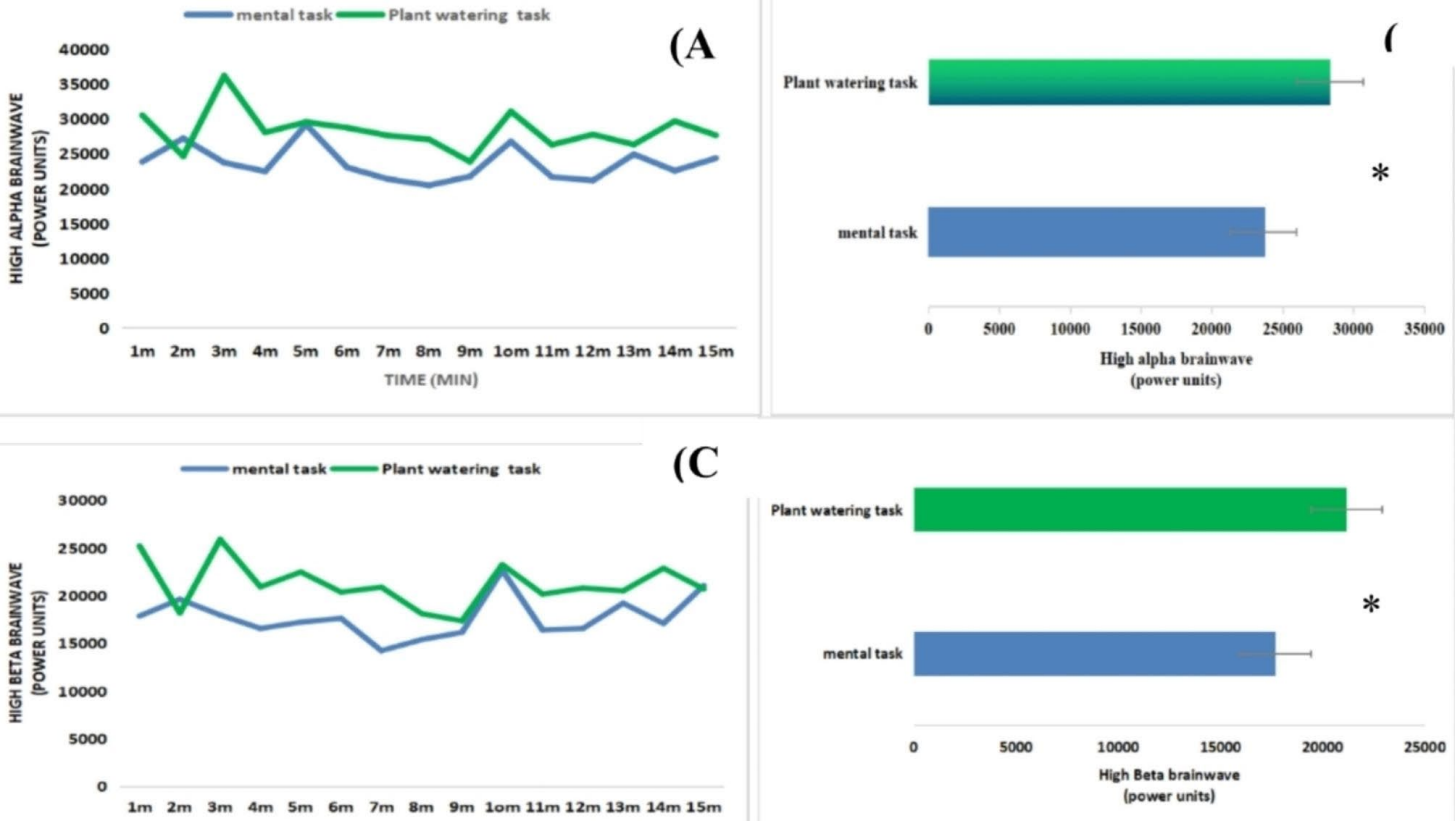
Comparison of EEG brainwave activity during the plant watering task and mental task (control) for 15 min. (**A**) High alpha brainwave activity 1–15 min between the two groups; (**B**) comparison of the overall mean power units values of high alpha brainwave between the two groups; (**C**) high beta brainwave activity 1–15 min between the two groups; (**D**) comparison of the overall mean power units values of high beta brainwave. N = 50; mean ± SD; *P < 0.05, paired t-test

After completing the horticultural task, the participants’ mean systolic (p = 0.01) and diastolic (p = 0.04) blood pressure values were considerably lower than those measured after completing the mental task (Fig. 2A). In addition, as shown in Fig. 2B, no discernible change in pulse rate (p = 0.89). Significant differences were observed in the high alpha and beta brainwaves (representing electrical brain activity) of both groups. When participants began the gardening task, the majority of the high alpha brainwaves’ mean power unit values were greater during the 1-minute analysis (Fig. 3a). According to the paired-samples t-test, the difference between the two tasks was significant (Fig. 3b; gardening task, 28326.92 ± 2956.05; mental task, 23618.74 ± 2501.16). A repeated-measures analysis of variance comparing the mean power units of high alpha brainwaves during the mental and gardening tasks in relation to time changes showed a significant difference in relaxation between the two tasks (F1,48 = 9.01; p = 0.004). Additionally, no discernible main impact for time was found within the groups (F14,48 = 1.61; p = 0.07). During the 1-minute analysis, the mean high beta power unit values of participants performing the gardening task were higher than those performing the mental task, and the paired-samples t-test showed that the difference between the two tasks was significant (Fig. 3d; gardening task, 21153.54 ± 2433.80; mental task, 17679.83 ± 2179.76; p < 0.01). A repeated-measures analysis of variance showed a significant difference (F1,48 = 4.07; p = 0.04) between the two activities when the high beta brainwave mean power unit values were compared (Fig. 3c). Furthermore, no discernible main effect of time on the groups was observed (F14,48 = 1.64; p = 0.06). The STAI and SDM self-reported questionnaires were completed and evaluated both before and after the tasks. The results showed a clear distinction between the two groups: after completing the gardening task, the participants’ anxiety mean scores, as measured with the STAI, were considerably lower than those obtained after completing the mental task (gardening task, 40.9 ± 4.14; mental task, 44.1 ± 2.72; p = 0.02). However, no discernible difference was found among the baseline values of the participant responses. Participants felt more at ease, natural, and comfortable after completing the gardening task than they did after completing the mental task (p < 0.01).

## Discussion

We set out to investigate whether performing a gardening chore elicits any calming effects by examining the psychological and physiological reactions of participants to watering indoor plants for 15 min. These responses were compared with those measured after performing a mental task that involved using a computer. Our study found that participants’ psychological and physiological reactions to the two activities were significantly different. As evidenced by the participants’ self-reported psychological reactions and measured physiological signs, watering indoor plants had noticeable relaxing effects. The physiological tests demonstrated that participants’ systolic and diastolic blood pressures dropped much higher after executing the gardening job than after conducting the mental exercise. This reduction indicated that significant alterations in blood pressure were observed following completion of the task. However, the pulse rate did not change significantly. An earlier investigation, in which participants participated in an HA that involved transplanting indoor plants, reported similar outcomes [38]. Compared to activities that exclude nature-based practices, carrying out activities that include such practices produce a stress-reducing effect by lowering blood pressure [39]. Gardening activities involving plant transplantation reduce stress, according to Hassan et al. [40]. Participants’ systolic and diastolic blood pressures decreased while watering indoor plants, clearly indicating a positive impact on their autonomic nerve systems. Blood pressure is directly influenced by the parasympathetic and sympathetic nervous systems. The results of this study show that watering indoor plants made individuals feel more at ease, less stressed, and happier.

We used electroencephalography to investigate why watering houseplants reduces tension, what role electrical brainwave activity plays in the two diseases, and how high alpha and beta brainwaves differ when watering plants. We discovered that both test environments changed the participants’ brain activity. For instance, an increase in high alpha and beta brainwave power units was observed throughout the gardening task, indicating that participants were experiencing tranquility and focus [41]. Additionally, our findings showed that, following the gardening task, not the mental task, high alpha brainwave activity increased more, which suggests less stress and great relaxation [42]. In other words, compared to the mental task, watering indoor plants resulted in a strong positive attitude and increased brain activity. A lower level of high beta brainwave activity during the mental task indicated weaker focus; however, our data showed an increase in high beta brainwave activity, demonstrating that individuals were more active and aware. High beta brainwave activity is observed when a person is extremely busy, whereas low beta brainwave activity is observed when they are sleepy. EEG devices use brainwave activity as a form of neurophysiological marker to identify the active human brain. Normally, the brain shows electrical activity during both periods of rest and daily activities (such as thinking, observing, and physical exercise) [43]. Every human produces brainwave activity early in the morning after waking up or before going to sleep, which is an interesting observation [44]. The actual manifestations of these electrical brainwave processes are our emotions and behaviors.

The SDM and STAI self-rated anxiety scores showed that the gardening task had a favorable impact on participants’ emotional states while the mental task had a negative impact. This study, in our opinion, offers insightful information on the effects of various surroundings and advances our understanding of brainwave patterns. Based on our findings, we strongly advise that healthy gardening-based activities be incorporated into strategies for managing mental health disorders.

Recognizing the limits of our study is crucial. First, our sample included only healthy Chinese college students; this may restrict the applicability of the findings to other populations. Second, we concentrated on a single variety of indoor plant, which might not accurately reflect the potential variability in effects produced by using different plant species. The limits of this study emphasize the need for additional research to provide a more thorough and practical understanding of the therapeutic effects of gardening-based activities on mental health. Future studies should examine how watering plants affects the blood pressure and brainwave activity of people of different ages who live in metropolitan areas and office employees to determine whether any differences may arise. Future studies should include participants undergoing stress induction tests before they enter the study setting to compare stress reduction effects; setting up a blank control group to achieve a baseline physiological measurement would be required.

## Conclusions

Our study provides strong empirical support for the claim that watering houseplants for 15 min significantly relaxes young Chinese people’s bodies and minds. This study highlights the importance of including outdoor activities, such as gardening, in developed spaces to enhance well-being. These discoveries could be used by landscape architects to include greenery and engaging gardening components in designs to provide calming, revitalizing settings that prioritize people’s mental and physical health. Landscape architecture that acknowledges the therapeutic effects of activities such as watering indoor plants may contribute to the development of sustainable and human-centered spaces that encourage a closer connection with nature, thereby boosting the general quality of life.

## Data Availability

The research data used to support the study’s findings have not been made public due to confidentiality concerns.
